# Validation of the Drug Abuse Screening Test (DAST-10): A study on illicit drug use among Chinese pregnant women

**DOI:** 10.1038/srep11420

**Published:** 2015-06-19

**Authors:** Lap Po Lam, Wing Cheong Leung, Patrick Ip, Chun Bong Chow, Mei Fung Chan, Judy Wai Ying Ng, Chu Sing, Ying Hoo Lam, Wing Lai Tony Mak, Kam Ming Chow, Robert Kien Howe Chin

**Affiliations:** 1 Department of Obstetrics and Gynaecology, Princess Margaret Hospital, Laichikok, Hong Kong SAR; 2 Department of Obstetrics and Gynaecology, Kwong Wah Hospital, 25 Waterloo Road, Yau Ma Tei, Kowloon, Hong Kong SAR; 3 Department of Paediatrics & Adolescent Medicine, Queen Mary Hospital, 102 Pokfulam Road, Hong Kong SAR; 4 Department of Paediatrics & Adolescent Medicine, Princess Margaret Hospital, Laichikok, Hong Kong SAR; 5 Hospital Authority Toxicology Reference Laboratory, Princess Margaret Hospital, Laichikok, Hong Kong SAR

## Abstract

We assessed the Chinese version of the Drug Abuse Screening Test (DAST-10) for identifying illicit drug use during pregnancy among Chinese population. Chinese pregnant women attending their first antenatal visit or their first unbooked visit to the maternity ward were recruited during a 4-month study period in 2011. The participants completed self-administered questionnaires on demographic information, a single question on illicit drug use during pregnancy and the DAST-10. Urine samples screened positive by the urine Point-of-Care Test were confirmed by gas chromatography-mass spectrometry. DAST-10 performance was compared with three different gold standards: urinalysis, self-reported drug use, and evidence of drug use by urinalysis or self-report. 1214 Chinese pregnant women participated in the study and 1085 complete DAST-10 forms were collected. Women who had used illicit drugs had significantly different DAST-10 scores than those who had not. The sensitivity of DAST-10 for identify illicit drug use in pregnant women ranged from 79.2% to 33.3% and specificity ranged from 67.7% to 99.7% using cut-off scores from ≥1 to ≥3. The ~80% sensitivity of DAST-10 using a cut-off score of ≥1 should be sufficient for screening of illicit drug use in Chinese pregnant women, but validation tests for drug use are needed.

Illicit drug use during pregnancy causes significant adverse obstetric and fetal outcomes, and is becoming an increasing major public health concern worldwide^1^,^2^,^3^,^4^,^5^,^6^,^7^. Early identification of illicit drug use by pregnant women during their antenatal visits would allow timely intervention to improve the perinatal outcomes^8^,^9^. Self-report measures are, by far, the most commonly used method of gathering information about drug use because they are cheap, easy to administer, and widely accessible^10^. The self-reported information could inform healthcare providers about the use of certain hazardous substances and can cover periods of drug use exceeding the detection window of urinary drug screening^11^. However, under-reporting of drug use may occur because respondents could fear possible negative social consequences or being stigmatized^12^,^13^. Also, compulsory routine urine drug testing in clinical settings such as antenatal clinics may raise certain ethical issues^14^ and may not be cost effective. The Drug Abuse Screening Test (DAST) is a patient self-administered tool, which has been designed as a brief instrument for clinical and non-clinical screening to detect potential drug abuse, which excludes alcohol and tobacco, during the past 12 months^15^. However such self-reported screening measures were found to be lacking in Chinese populations.

In light of the alarming rise in illicit drug use in Chinese populations and lack of effective screening tools, we validated the Chinese version of Drug Abuse Screening Test (DAST-10) to detect illicit drug use among low risk Chinese pregnant women. We studied the psychometric properties of DAST-10 and examined its use in identifying illicit drug use during pregnancy and in assessing the degree of related problems.

## Results

A total of 1483 Chinese pregnant women from Hong Kong were recruited during the 4-month study period, 1416 were attending their first antenatal visit and 67 were visiting the maternity ward for the first time. Overall, 1214 pregnant women participated in this study (81.9% response rate) and 1214 DAST-10 (Chinese version) screening forms were collected. Demographic characteristics are shown in Table 1. Among the 1214 DAST-10 forms collected, 129 forms were not complete. Among the 1085 completed forms, the scores ranged from 0 to 8, with 66.6% scoring 0, 30.1% scoring 1, 2.2% scoring 2, and the remaining scoring from 3 to 8. The median DAST score was 0 (interquartile range 0 to 1) among all the participants. The overall internal consistency was 0.64.

Among the 1085 women with completed DAST-10 forms, 3 urine samples were lost or spilt before transfer to laboratory for test. 13 urine samples were confirmed to be positive for one or more of the illicit drugs tested. Among the 1069 urine samples which screened negative by the POCT cards, 42 urine samples were randomly selected as negative controls for confirmatory GC-MS, which were all confirmed to be negative for any drugs.

In the anonymous questionnaires collected, one woman did not answer the single question on illicit drug use during current pregnancy, 14 women admitted to a history of illicit drug use during pregnancy and 1070 women denied a history of illicit drug use during pregnancy. A total of 24 pregnant women had either positive urinalysis for drug use or self-reported a history of illicit drug use.

### Principal component analysis

A principal component analysis (PCA) was conducted on the 10 items of the DAST-10 using oblique rotation (direct oblimin). The Kaiser-Meyer-Olkin measure (KMO = 0.688) verified the sampling adequacy for the analysis. Barlett’s test of sphericity X^2^ (45) = 1472.30 (P < 0.001) indicated the correlations between items were sufficiently large for PCA. Eigenvalues were calculated for each component in the data. Three components had eigenvalues over Kaiser’s criterion of 1 and in combination could explain 50.2% of the variance. Table 2 shows the factor loadings after rotation.

### Criterion and concurrent validity

The DAST-10 scores of pregnant women were significantly different between those who had and those who had not used illicit drugs.

Using urinalysis as a measure of illicit drug use (Measure 1), 13 pregnant women had positive urinalysis with a median DAST score of 1 (interquartile range 0 to 2), whereas 1069 women had negative urinalysis with a median DAST score of 0 (interquartile range 0 to 1; Mann–Whitney U test z = –3.39, P = 0.001). There was significant correlation between DAST-10 scores and the results of urinalysis (r = 0.103, P = 0.001, two tailed).

Using the self-reported single question as a measure of illicit drug use (Measure 2), 14 pregnant women self-reported illicit drug use during pregnancy with a median DAST score of 3 (interquartile range 1 to 5), whereas 1070 women reported no illicit drug use during pregnancy with a median DAST score of 0 (interquartile range 0 to 1; Mann–Whitney U test z = –6.64, P < 0.001). There was significant correlation between DAST-10 scores and the self-reported drug use (r = 0.202, P < 0.001, two tailed).

Using urinalysis-or-self-reported illicit drug use during pregnancy (Measure 3) as measure of illicit drug use, 24 pregnant women showed evidence of illicit drug use with a median DAST score of 2 (interquartile range 1to 3), whereas 1061 women had no history of illicit drug use with a median DAST score of 0 (interquartile range 0 to 1; Mann–Whitney U test z = –6.31, P < 0.001). There was significant correlation between DAST-10 scores and evidence of drug use by Measure 3 (r = 0.192, P < 0.001, two tailed).

The DAST-10 scores also significantly correlated with past history of illicit drug use before pregnancy (r = 0.141, P < 0.001, two tailed) and with current cigarette smoking (r = 0.061, P = 0.047, two tailed).

### Face validity

**Figure 1:**
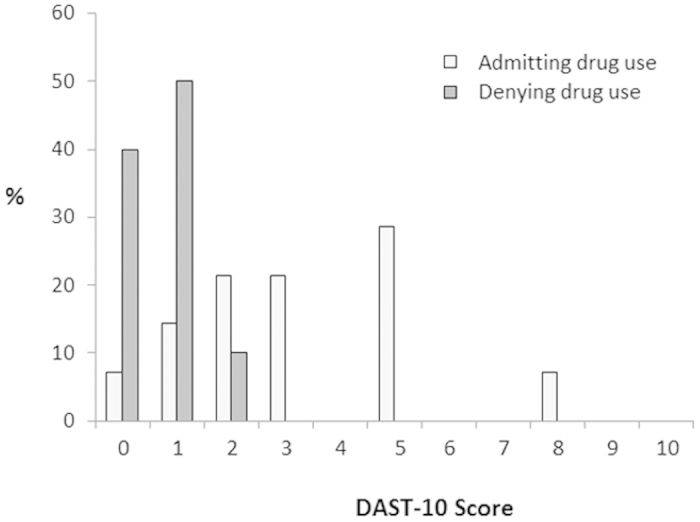
Distribution of pregnant women admitting and denying current drug use (y axis) versus the total DAST-10 score (x-axis) for the detection of illicit drug use among pregnant women.

The DAST-10 (Chinese version) had high face validity. Among the pregnant women with evidence of illicit drug use during pregnancy, those who denied current illicit drug use during pregnancy had significantly lower DAST scores (Spearman’s rho correlation coefficient = –0.67, P < 0.001, two-tailed) compared to those who admitted current illicit drug use. (Fig. 1)

### Discriminative validity

**Figure 2:**
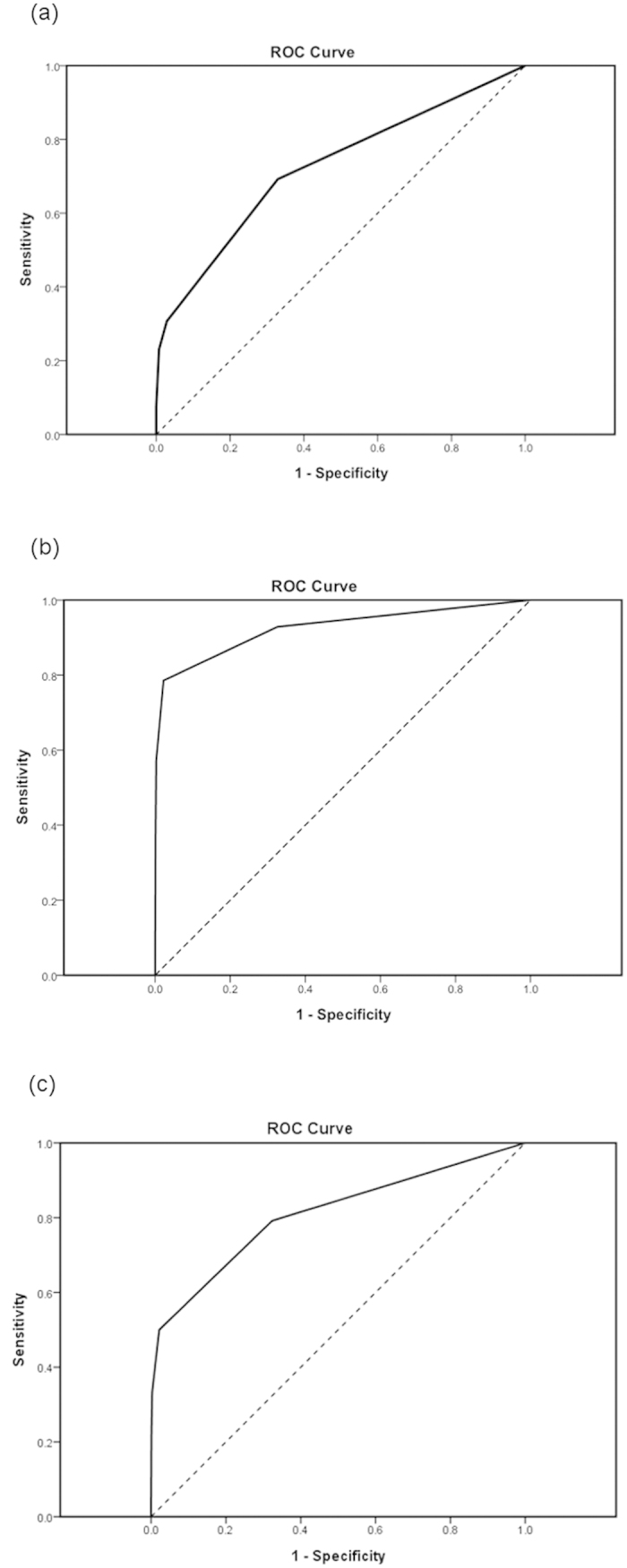
The ROC curve for DAST-10 detection of (**a**) positive urinalysis for illicit drug use (AUROC = 0.73; 95% CI, 0.56–0.89; asymptotic significance P = 0.005); (**b**) self-reported illicit drug use (AUROC = 0.93; 95% CI, 0.83–1.00; asymptotic significance P < 0.001); and (**c**) evidence of illicit drug use (AUROC = 0.81; 95% CI, 0.70–0.92; asymptotic significance P < 0.001).

Tables 3, 4, 5 show the corresponding sensitivity, specificity, positive predictive value, negative predictive value and overall accuracy of the different DAST-10 cut-off scores. The AUROC was 0.73 for DAST-10 (95% CI, 0.56 to 0.89, asymptotic significance P = 0.005) detection of positive urinalysis for illicit drug use (Fig. 2a). The AUROC was 0.93 for DAST-10 (95% CI, 0.83 to 1.00, asymptotic significance P < 0.001) detection of self-reported illicit drug use during pregnancy (Fig. 2b). The AUROC was 0.81 for DAST-10 (95% CI, 0.70 to 0.92, asymptotic significance P < 0.001) detection of evidence of illicit drug use during pregnancy (Fig. 2c).

### Degree of problems related to illicit drug use

**Figure 3:**
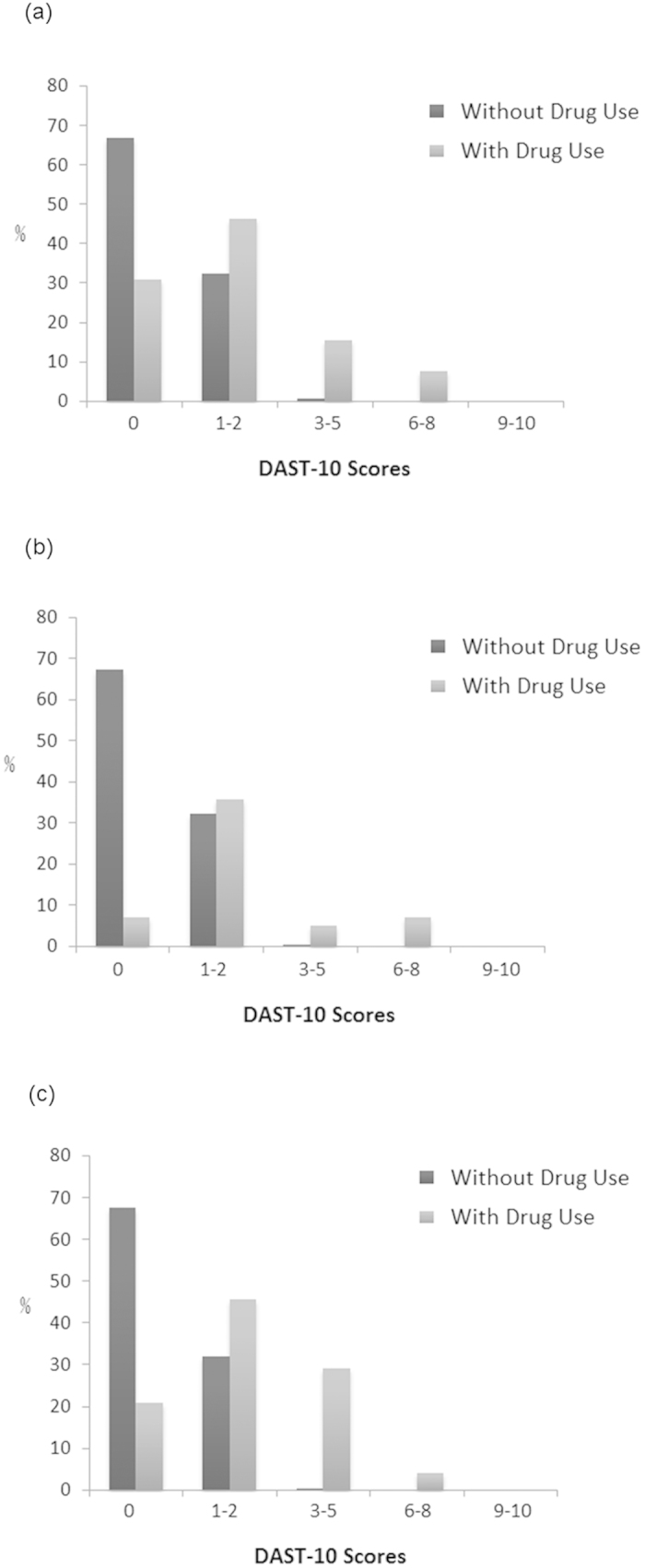
Distribution of pregnant women with and without drug use (y axis) versus the five categories of DAST-10 scores (x axis) according to the degree of problems (0: No problem; 1–2 Low level; 3–5 Moderate level; 6–8 Substantial level; 9–10 Severe level) for (**a**) urinalysis for illicit drug (Measure 1), (**b**) self-reported illicit drug use (Measure 2), and (**c**) evidence of drug use during pregnancy by urinalysis or self-report (Measure 3).

The distribution of participants in the five categories of DAST-10 scores according to the degree of problems (0: No problem; 1–2 Low level; 3–5 Moderate level; 6–8 Substantial level; 9–10 Severe level)^16^ are shown in Fig. 3. Among the pregnant women who used illicit drugs, the degree of problems shown by DAST-10 was significantly correlated with past history of illicit drug use before pregnancy (r = 0.458, P = 0.024, two tailed) and with a criminal history (r = 0.446, P = 0.033, two tailed), but there was no significant correlation with current cigarette smoking, alcohol drinking, gambling, domestic violence, psychiatric illness, sexually transmitted diseases or thrombosis.

## Discussion

Illicit drug use is an important public health problem worldwide. In Hong Kong, a survey showed that 1.6% of female students had history of illicit drug use^17^. For pregnant women, early identification of illicit drug use during their antenatal visits would allow timely intervention to improve the perinatal outcomes^8^,^9^. In clinical settings including antenatal clinics, compulsory use of routine urine drug testing may raise certain ethical issues^14^ and may not be cost effective. Several instruments for substance abuse screening have been validated for use during pregnancy^14^. DAST-10 is a relatively brief tool for identifying potential drug-related problems^15^ and has been recommended as an assessment tool for use in general medical settings^18^. One study showed that DAST-10 used in a primary care setting had 100% sensitivity (95% CI:90.6%–100%) and 77% specificity (95% CI: 71.5%–81.9%) using a cut-off ≥3 for the detection of problems related to current drug use^19^. Other studies showed a range of sensitivity (95% to 41%) and specificity (42% to 99%) levels for different cut-off scores for DAST-10 used in the screening of psychiatric patients by structured interviews^20^,^21^,^22^. Our study on DAST-10 (Chinese version) also showed comparable results of a sensitivity of 92.9% and specificity of 67.4% for a cut-off score ≥1 when using self-reported drug use during pregnancy as the gold standard (Measure 2). The gold standards (criterion diagnosis) used in the above mentioned studies were self-reported structured interviews. It is possible that the respondent may deny drug use in the self-report question as well as in the DAST-10 screening. Hence, the validity of the screening in this situation may be falsely inflated^23^ by comparisons with gold standards based on such measures, resulting in high accuracy values in the above mentioned studies^19^,^20^,^21^,^22^. Furthermore, there was an inherent recall bias and report bias (mainly under-reporting) while filling in the DAST-10 screening form and answering the single question on illicit drug use.

Therefore, the use of bioanalytical measures as the gold standard for studying the validity of the screening tool would be more objective^23^. In our study, the use of urinalysis (Measure 1) could detect those pregnant women who did not report their current drug use and overcome the issue of under-reporting. And with the utilization of GC-MS, false positive results were eliminated. The sensitivity was 69.2% and specificity was 70.0% using a cut-off score ≥1, and was 30.8% and 97.1%, respectively, using cut-off score ≥2. In the literature, a study on 274 high risk postpartum women (mainly low income African-American) examined the ability of DAST-10 to identify prenatal drug use using hair and urine samples as criterion variables^10^. The sensitivity was 47% and specificity was 82% using a cut-off score ≥1, and was 23% and 94%, respectively, using cut-off score ≥2. Another study^13^ on 400 low-income, post-partum African American women showed an even lower sensitivity of 37% and specificity 83% using a cut-off score ≥1, and sensitivity 7% and specificity 97% using a cut-off score ≥2. Due to the low sensitivity, it was suggested that the DAST-10 may have limited utility for pregnant women^10^.

However, using just either one of the methods (urinalysis or self-report drug use) as gold standards has limitations. In fact, the two methods are complementary to each other. Bioanalysis alone is not an ideal measure of illicit drug use. It may fail to detect drug use as it can only detect drug levels above a specific cut-off level within a specific period of time after drug use^1^. Negative results would be shown if the drug use is beyond the detection window (e.g. intermittent use)^14^, if the drug level in the sample is lower than the detection cut-off level, or if the drugs used are not included in the bioanalysis screening tests. The inclusion of self-report measure could, therefore, complement any limitations in the urinalysis, and is useful to detect the cases when illicit drug use is beyond detection by urinalysis. The evidence of drug use by urinalysis or self-report (Measure 3) detects drug use when EITHER urinalysis OR self-reported drug use is positive, as each method detects different types of pregnant women with illicit drug use of different patterns^14^,^24^. The results obtained by Measure 3 would be closer to the genuine situation than applying only one of the measures. Using Measure 3, the sensitivity was 79.2% using a cut-off score ≥1 and was 50% using a cut-off score ≥2, which was higher than using bioanalysis alone, and was also higher than reported by previous studies^10^,^13^.

Our study on DAST-10 (Chinese version) was performed on more than 1200 low risk pregnant women in a general antenatal clinic setting in a Chinese population, which was one of the largest study to date, compared to previous studies conducted on vulnerable populations (mainly low-income, postpartum women, African-American women)^10^,^13^. Furthermore, anonymity encouraged the pregnant women to participate and provide information without fear of being identified. The use of urinalysis was objective and could overcome the inherent bias of under-reporting, and the additional use of self-report question on drug use could complement the limitation of urinalysis, which was a strength in this validation study.

The internal consistency in our study was 0.64, which was low compared to other studies^25^. In studies on drug abuse screening of patients with psychiatric disorders, the coefficient alpha ranged from 0.86 to 0.94^20^,^22^. In a study validating DAST-10 among African American pregnant women^10^ the coefficient alpha was 0.62, which was similar to the value obtained in our study, and is in the acceptable range for screening.

Among 1483 invited participants, total 1214 questionnaires were collected (response rate 81.9%). As participation was anonymous and volunteer, the data and background information of the 269 non-participants was lacking. And because our study was anonymous in its design, test-retest reliability could not be performed. The information of gestational age of women at the time of study was not included in the questionnaire, and the recall time period was not available for analysis. Nevertheless, the majority (99.9%) of the respondent were recruited from out-patient clinic at their first antenatal visits. In our antenatal clinic, around 70–80% of pregnant women were less than 20 weeks of gestation at their first antenatal visits. We also included the unbooked pregnant women attending the maternity ward for the first time because those with illicit drugs may have had no previous antenatal care^5^. As illicit drug use is illegal in Hong Kong, the participants were unlikely to recall incorrectly on their history of drug use while answering the single-question unless they deliberately denied such history. Our study showed that pregnant women who denied using illicit drugs during pregnancy tended to report lower DAST scores, which was also the case in a study by Skinner^15^. Because DAST has high face validity, it is susceptible to the respondents giving false answers to try to hide (drug-related) problems.

In light of the alarming rise in illicit drug use in Chinese populations and lack of effective screening tools, our study is the first to validate in the literature the Chinese version of DAST-10 to detect illicit drug use among low risk Chinese pregnant women. As it has not ever been applied to any other independent groups (e.g. general population, men, non-pregnant women) of Chinese population, the comparison of our results with these groups is not possible. However the results in our study in assessing the DAST-10 (Chinese version) for identifying illicit drug use among Chinese pregnant women were comparable to the previous studies on validation of DAST-10 (English version) on detection of drug use among pregnant women in other population^10^,^13^,^20^,^21^,^22^. The Chinese version of the DAST-10 provides a viable option for clinicians to screen pregnant women for illicit drug use and to assess drug-related problems in Chinese populations. Using a cut-off score of ≥1, the sensitivity in our study approached 80%, which could be sufficient for screening of illicit drug use in pregnancy among Chinese population, but further confirmatory measures are needed to confirm the status of drug use.

## Methods

### Drug Abuse Screening Test (DAST)-10

The DAST was developed and validated by Dr. Harvey A. Skinner at the Addiction Research Foundation, Toronto, Canada (now the Center for Addiction and Mental Health). The DAST is copyrighted by Dr. Harvey A. Skinner, although it has been made available for non-profit research, clinical, and training purposes. Permission for the use of DAST-10 in this study was obtained from the developer. The DAST-10 is a 10-item, yes/no, self-report instrument that has been shortened from the original 28-item DAST^16^. Each question answered with a “Yes” scores 1 point, except for Question 3, which scores 1 point for “No”. All items assess drug use in general, without referring to specific types of drugs. The words “drug use” and “drug abuse” used in the form refers to the use of any non-prescription drugs or the use of any prescribed or over-the-counter medications used in excess of the recommended doses. The questionnaire does not include any items on alcohol or tobacco.

**Figure 4:**
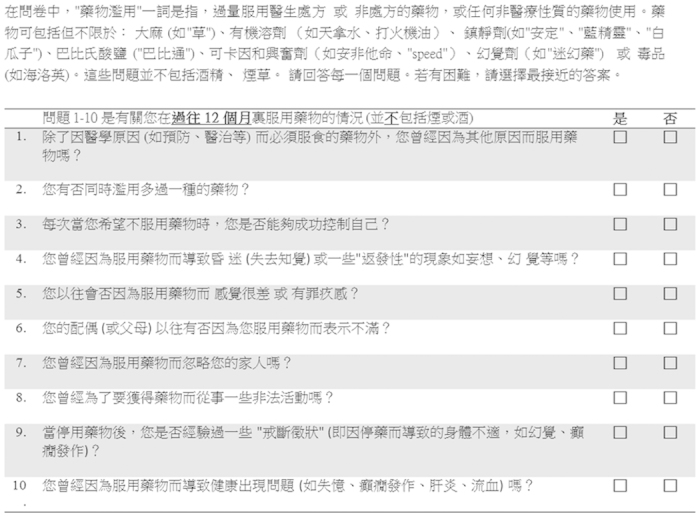
A sample of the Drug Abuse Screening Test (DAST-10) Chinese Version. An English translation can be found in the Supplementary Information.

The Chinese version of the DAST-10 (Fig. 4) was first translated from English into Chinese by the first author and then back translated by an independent researcher with postgraduate qualifications (Supplementary Note S1). Both translators are bilingual. Variations between direct- and back-translations were discussed among authors and modified accordingly. The modified Chinese version was also sent to different obstetricians for comments before being used in the study. The Chinese version of the DAST-10 questionnaire was made available to Chinese pregnant women attending the antenatal clinic, who could choose to complete the form anonymously. Information and instructions on the use of DAST-10 were given to the pregnant women before filling in the form.

### Questionnaire

In the questionnaire, they were also asked directly whether they were currently using illicit drugs during their pregnancy, using the question 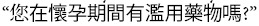 (translated into English as “Have you used any illicit drugs during your pregnancy?”).

### Point-of-Care Testing (POCT) of Urine

The *FirstSign*^*TM*^ *One Step Multi Drug Screen (MDS-10) Cards* and *FirstSign*^*TM*^ *One-Step Drug of Abuse Test Multi-drug (W.H.P.M., Inc.)* were used to test the collected urine samples for the presence of the following drugs: Amphetamine (AMP), Barbiturate (BAR), Benzodiazepine (BZD), Cocaine (COC), Ketamine (KET), Methylenedioxymethamphetamine (MDMA/Ecstasy), Methadone (MTD), Methamphetamine (MAMP), Opiates (OPI), and Cannabinoids (THC). These drugs are the most commonly used drugs among adult drug users in Hong Kong^26^. The two assays are one-step lateral flow chromatographic immunoassays for qualitative determination of drug substances in human urine specimens based on the principle of competitive binding. Positive results are given if the urine contains any of the corresponding substances in amounts above the cut-off concentration. Table 4 shows the cut-off concentrations for each drug tested by the immunochemical assays^27^. These cut-off levels were deliberately selected to reflect significant exposure from substance use rather than accumulated environmental exposure.

### Confirmatory test

The POCT immunoassay urine tests are sensitive^28^ but prone to cross reactivity^27^,^29^. The presence of commonly used medications, including chlorpheniramine and promethazine, could give rise false positives^27^. To ensure the quality of the study, validation tests were performed using Gas-chromatography-mass spectrometry (GC-MS) to confirm the presence of the drug. Urine samples screening negative by the POCT cards were randomly selected for GC-MS analysis as the negative controls.

### Study population

The study was conducted in two major public hospitals (each with approximately 5000 deliveries per year) in Hong Kong, Kwong Wah Hospital from May 2011 to June 2011 and Princess Margaret Hospital from September 2011 to October 2011. Local Chinese pregnant women newly attending the antenatal clinics and local unbooked Chinese pregnant women presenting to the maternity ward for the first time were recruited for the study. Pregnant women who were not Hong Kong residents were excluded from the study.

After routine urine testing for albumin and glucose, the pregnant women were asked to provide their urine samples. The urine sample, questionnaire with direct questions on illicit drug use during pregnancy, and the self-administered DAST-10 (Chinese version) screening form from the same pregnant woman were anonymously labeled with the same unique number. The DAST-10 scores were calculated from the DAST-10 screening forms. The urine samples collected were tested by POCT and were performed by a trained staff who was blinded from the results of the DAST-10 screening and the results of the single question on self-reported drug use. Urine samples screened positive by the POCT were sent to a laboratory for confirmatory testing by gas chromatography-mass spectrometry. The laboratory staff performing the confirmatory tests was also blinded from the urine POCT results and all other results from DAST-10 or the self-report drug use.

### Gold standards (Criterion diagnosis)

Different measures were used as the gold standards for detection of illicit drug use and to assess the performance of DAST-10 in identifying illicit drug use during pregnancy. (Measure 1) Urinalysis: positive urinalysis results were urine samples that tested positive by the POCT assay confirmed by GC-MS, whereas negative urinalysis results were urine samples that tested negative by the POCT assay or tested negative by GC-MS. (Measure 2) Self-reported drug use: positive results were those pregnant women who self-reported illicit drug use during pregnancy, whereas negative results were those who reported no illicit drug use during pregnancy. (Measure 3) Evidence of drug use by urinalysis or self-report: positive results were those who were positive for either the urinalysis or the questionnaire result, whereas negative results were those who were negative for both the urinalysis and questionnaire result.

Details of the study were explained to the pregnant women by the nurses in the antenatal clinic or maternity ward, and the women were given an information sheet about the study. The pregnant women were free to choose whether to leave the urine sample or to fill in the anonymous questionnaires and DAST-10 screening form. Informed consent was obtained from all participants. Methods in the study were carried out in accordance with the approved guidelines of the Clinical Research Ethics Committee of the Hong Kong Hospital Authority. Ethics approval was obtained from the Clinical Research Ethics Committee (Kowloon West Cluster) of the Hong Kong Hospital Authority.

### Statistical analysis

Mann–Whitney U test was used to compare the difference of total DAST-10 scores between participants with and without illicit drug use for each of the measures. Spearman’s rho correlation coefficients were obtained from bivariate correlation tests. Receiver Operating Characteristic (ROC) curve data, sensitivity, specificity, positive predictive value (PPV), negative predictive value (NPV) and overall accuracy with different cut-off scores of DAST-10 were calculated. Sensitivity refers to the true positive rate (i.e., the number of women scoring higher or equal to the cut-off score of DAST out of the number of those with positive results for illicit drug use). Specificity refers to the true negative rate (i.e., the number women scoring lower than the cut-off score out of the number of those with negative results for illicit drug use). PPV is defined as the proportion of true cases that were correctly identified by DAST-10 (the proportion of women scored higher or equal to the cut-off score of DAST having positive results for illicit drug). NPV is defined as the proportion of pregnant women with negative result for illicit drug use who were correctly identified by DAST-10 (the proportion of women scored lower than the cut-off score of DAST having negative results for illicit drug). Overall accuracy is defined as the number of true positives and true negatives among the total number of women undergoing the tests. For the ROC curves for DAST-10, test sensitivity vs. (1 – specificity) for each cut-off point was plotted. The area under the curve (AUROC) was calculated as a measure of overall test performance, with a score of 1.0 representing a perfect test and a score of 0.5 a test that provided no information.

## Additional Information

**How to cite this article**: Lam, L. P. *et al.* Validation of the Drug Abuse Screening Test (DAST-10): A study on illicit drug use among Chinese pregnant women. *Sci. Rep.* **5**, 11420; doi: 10.1038/srep11420 (2015).

## Supplementary Material

Supplementary Information

## Figures and Tables

**Table 1:** Participant demographic characteristics (N = 1214).

**Age**	**31.0**
Gravidity	2 (1–3)
Parity - live birth	0 (0–1)
Number of previous terminations of pregnancy (TOPs)	0 (0–1)
On public financial assistance	2.4%
Single	7.9%
Unplanned pregnancy	23.5%
Smoking before pregnancy	15.7%
Smoking during pregnancy	1.4%
Drinking before pregnancy	4.1%
Drinking during pregnancy	0.1%
Illicit drug use before pregnancy	3%

Age is presented as the mean, while gravidity, parity and number of TOPs are presented as the median and interquartile (25th–75th percentile) range.

**Table 2:**
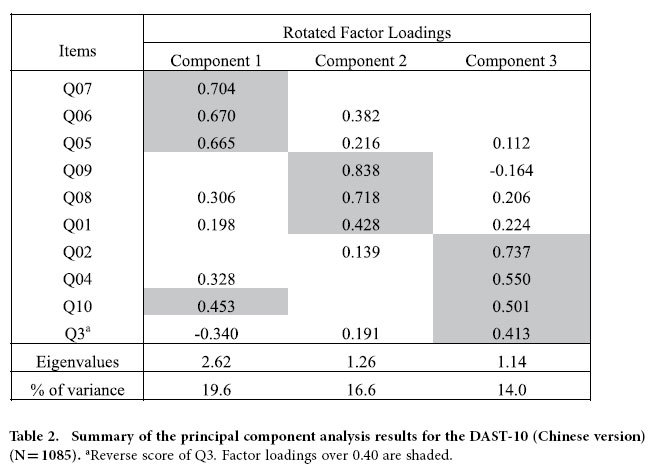
Summary of the principal component analysis results for the DAST-10 (Chinese version) (N = 1085).

**Table 3:** Sensitivity, specificity, positive predictive value, negative predictive values and overall accuracy of the different DAST-10 cut-off scores for detection of positive urinalysis (Measure 1).

**DAST cut-off score**	**Sensitivity**	**Specificity**	**Positive predictive value**	**Negative predictive value**	**Overall accuracy**
≥1	69.2%	70.0%	2.4%	99.4%	67%
≥2	30.8%	97.1%	11.4%	99.1%	96.3%
≥3	23.0%	99.3%	27.3%	99.1%	98.3%

**Table 4:** Sensitivity, specificity, positive predictive value, negative predictive values and overall accuracy of the different DAST-10 cut-off scores for detection of self-reported illicit drug use during pregnancy (Measure 2).

**DAST cut-off score**	**Sensitivity**	**Specificity**	**Positive predictive value**	**Negative predictive value**	**Overall accuracy**
≥1	92.9%	67.4%	3.6%	99.9%	67.7%
≥2	78.6%	97.8%	31.4%	99.7%	97.5%
≥3	57.1%	99.7%	72.7%	99.4%	99.2%

**Table 5:** Sensitivity, specificity, positive predictive value, negative predictive values and overall accuracy of the different DAST-10 cut-off scores for the detection of evidence of drug use by urinalysis or self-report (Measure 3).

**DAST cut-off score**	**Sensitivity**	**Specificity**	**Positive predictive value**	**Negative predictive value**	**Overall accuracy**
≥1	79.2%	67.7%	5.2%	99.3%	67.9%
≥2	50.0%	97.8%	34.3%	99.9%	96.8%
≥3	33.3%	99.7%	72.7%	98.5%	98.2%

**Table 6:** The cut-off concentrations for each drug used in the immunochemical assays.

Amphetamine	1000 ng/ml of d-amphetamine
Barbiturate	300 ng/ml of secobarbital
Benzodiazepine	300 ng/ml of oxazepam
Cocaine	300 ng/ml of benzoylecgonine
Ketamine	1000 ng/ml of ketamine
MDMA (Ecstasy)	500 ng/ml of MDMA
Methadone	300 ng/ml of methadone
Methamphetamine	1000 ng/ml of D-methamphetamine
Opiate	300 ng/ml of morphine
Cannabinoid/Marijuana (THC)	50 ng/ml of 11-nor-Δ^9^-THC-9-COOH

## References

[b1] Narkowicz S., Plotka J., Polkowska Z., Biziuk M. & Namiesnik J. Prenatal exposure to substance of abuse: a worldwide problem. Environ Int 54, 141–163 (2013). 23454110 10.1016/j.envint.2013.01.011

[b2] Vucinovic M. *et al.* Maternal and neonatal effects of substance abuse during pregnancy: our ten-year experience. Yonsei Med J 49, 705–713 (2008). 18972589 10.3349/ymj.2008.49.5.705 PMC2615365

[b3] Kuczkowski K. M. The effects of drug abuse on pregnancy. Curr Opin Obstet Gynecol 19, 578–585 (2007). 18007137 10.1097/GCO.0b013e3282f1bf17

[b4] Ladhani N. N., Shah P. S. & Murphy K. E. Prenatal amphetamine exposure and birth outcomes: a systematic review and metaanalysis. Am J Obstet Gynecol 205, 219 e211–217 (2011). 10.1016/j.ajog.2011.04.016 21658669

[b5] Lam S. K., To W. K., Duthie S. J. & Ma H. K. Narcotic addiction in pregnancy with adverse maternal and perinatal outcome. Aust N Z J Obstet Gynaecol 32, 216–221 (1992). 1445130 10.1111/j.1479-828x.1992.tb01950.x

[b6] Miles D. R., Lanni S., Jansson L. & Svikis D. Smoking and illicit drug use during pregnancy: impact on neonatal outcome. J Reprod Med 51, 567–572 (2006). 16913548

[b7] Buckingham-Howes S., Berger S. S., Scaletti L. A. & Black M. M. Systematic review of prenatal cocaine exposure and adolescent development. Pediatrics 131, e1917–1936 (2013). 23713107 10.1542/peds.2012-0945 PMC3666107

[b8] Goler N. C., Armstrong M. A., Taillac C. J. & Osejo V. M. Substance abuse treatment linked with prenatal visits improves perinatal outcomes: a new standard. J Perinatol 28, 597–603 (2008). 18580882 10.1038/jp.2008.70

[b9] Kaltenbach K. & Finnegan L. Prevention and treatment issues for pregnant cocaine-dependent women and their infants. Ann N Y Acad Sci 846, 329–334 (1998). 9668419

[b10] Grekin E. R. *et al.* Drug use during pregnancy: validating the Drug Abuse Screening Test against physiological measures. Psychol Addict Behav 24, 719–723 (2010). 21198230 10.1037/a0021741 PMC3878301

[b11] Horrigan T. J., Piazza N. J. & Weinstein L. The substance abuse subtle screening inventory is more cost effective and has better selectivity than urine toxicology for the detection of substance abuse in pregnancy. J Perinatol 16, 326–330 (1996). 8915928

[b12] Magura S. & Kang S. Y. Validity of self-reported drug use in high risk populations: a meta-analytical review. Subst Use Misuse 31, 1131–1153, (1996). 8853234 10.3109/10826089609063969

[b13] Ondersma S. J. *et al.* Development and preliminary validation of an indirect screener for drug use in the perinatal period. Addiction 107, 2099–2106 (2012). 22882721 10.1111/j.1360-0443.2012.03982.x PMC3499681

[b14] Goodman D. J. & Wolff K. B. Screening for substance abuse in women’s health: a public health imperative. J Midwifery Womens Health 58, 278–287 (2013). 23631601 10.1111/jmwh.12035

[b15] Skinner H. A. The drug abuse screening test. Addict Behav 7, 363–371 (1982). 7183189 10.1016/0306-4603(82)90005-3

[b16] Bohn M. J., Babor T. F. & Kranzler H. R. Validity of the drug abuse screening test (DAST-10) in inpatient substance abusers: Problems of drug dependence. Proceedings of the 53rd Annual Scientific Meeting, The Committee on Problems of Drug Dependence, DHHS Publication No. 92–1888. NIDA Research Monograph 119, 233 (1991).

[b17] Government, H. K. S. A. R. The 2011/12 Survey of Drug Use among Students. Section 2 17 (Narcotics Division of Security Bureau, 2013).

[b18] Ghitza U. E. *et al.* Common data elements for substance use disorders in electronic health records: the NIDA Clinical Trials Network experience. Addiction 108, 3–8 (2013). 22563741 10.1111/j.1360-0443.2012.03876.x

[b19] Smith P. C., Schmidt S. M., Allensworth-Davies D. & Saitz R. A single-question screening test for drug use in primary care. Arch Intern Med 170, 1155–1160 (2010). 20625025 10.1001/archinternmed.2010.140 PMC2911954

[b20] Cocco K. M. & Carey K. B. Psychometric properties of the Drug Abuse Screening Test in psychiatric outpatients. Psychological Assessment 10, 408–414 (1998).

[b21] Maisto S., Carey M., Carey K., Gordon C. & Gleason J. Use of the AUDIT and the DAST-10 to identify alcohol and drug use disorders among adults with a severe and persistent mental illness. Psychological Assessment 12, 186–192 (2000). 10887764 10.1037//1040-3590.12.2.186

[b22] Carey K. B., Carey M. P. & Chandra P. S. Psychometric evaluation of the alcohol use disorders identification test and short drug abuse screening test with psychiatric patients in India. J Clin Psychiatry 64, 767–774 (2003). 12934976 10.4088/jcp.v64n0705 PMC2441940

[b23] Ondersma S. J. *et al.* The importance of indirect screening and objective gold standards: a response to Terplan (2012). Addiction 108, 1002 (2013). 23587087 10.1111/add.12131

[b24] Chasnoff I. J. *et al.* The 4P’s Plus screen for substance use in pregnancy: clinical application and outcomes. J Perinatol 25, 368–374 (2005). 15703775 10.1038/sj.jp.7211266

[b25] Yudko E., Lozhkina O. & Fouts A. A comprehensive review of the psychometric properties of the Drug Abuse Screening Test. J Subst Abuse Treat 32, 189–198 (2007). 17306727 10.1016/j.jsat.2006.08.002

[b26] Government, H. K. S. A. R. Central Registry of Drug Abuse Report Sixtieth Report. Narcotics Division, Security Bureau (2011). Available at: http://www.nd.gov.hk/pdf/report/crda_60th/crda_60th_full_report.pdf (Accessed: 1st September 2011).

[b27] Inc. W H P M. One Step Drug of Abuse Test. Package Insert for Multi Drug Screen Test. Available at: http://www.whpm.com/pdf/2.8.pdf (Accessed: 1st Septeber 2011).

[b28] Food-and-Drug-Administration, U. S. Center for Devices and Radiological Health (CDRH). 510(k) Substantial Equivalence Determination Decision Summary. (2004). Available at: http://www.accessdata.fda.gov/cdrh_docs/reviews/K032575.pdf (Accessed: 1st September 2011).

[b29] Moeller K. E., Lee K. C. & Kissack J. C. Urine drug screening: practical guide for clinicians. Mayo Clin Proc 83, 66–76 (2008). 18174009 10.4065/83.1.66

