# Supplementary material for: Validation of the Drug Abuse Screening Test (DAST-10): A study on illicit drug use among Chinese pregnant women
Source: Sci Rep. 2015 Jun 19;5:11420. doi: 10.1038/srep11420 (PMC4473689; doi:10.1038/srep11420)
Supplement: Supplementary Information [file srep11420-s1.pdf]

Supplementary Information

Title:

Validation of the Drug Abuse Screening Test (DAST-10) : A study on illicit drug use among Chinese pregnant women

Authors:

Lap Po Lam, Wing Cheong Leung*, Patrick Ip, Chun Bong Chow, Mei Fung Chan, Judy Ng, Chu Sing, Ying Hoo Lam, Wing Lai Tony Mak, Kam Ming Chow & Robert KH Chin

Supplementary Note S1. Back-translation of the Chinese version of DAST-10 screening form

In the questionnaire, the term "drug abuse" refers to overdose of prescribed medications or over-the-counter medications, and any non-medical use of drugs. Drugs may include, but are not limited to: cannabis (e.g. marijuana), organic solvents (e.g. thinner, gas), tranquilizers (e.g "white seeds"), barbiturates, cocaine and stimulants (e.g. amphetamines, "speed"), hallucinogens (e.g. "ecstasy") or narcotics (e.g. heroin). These questions do not include alcohol or tobacco. Please answer every question. If difficulties encountered, please select the one nearest to your answer.

Question 1-10 is about your condition of potential drug use (not including tobacco or alcohol drug) in the past 12 months.

1. Besides taking drugs for medical reasons, have you ever taken any drugs due to other reasons?

2. Have you abused more than one drug at the same time?

3. Are you able to control and stop yourself from using the drugs when you want to?

4. Have you experienced any loss of consciousness (blackouts), or delusions or hallucinations (flashbacks) due to drug use?

5. Do you ever feel bad or guilty for taking drugs?

6. Has your spouse (or parent) ever complained to you or expressed dissatisfaction on your drug use?

7. Have you neglected your family members because of your drug use?

8. Have you ever engaged in any illegal events in order to obtain drugs?

9. After stopping drugs, have you experienced any withdrawal symptoms (e.g. physical discomfort, hallucinations, seizures)?

10. Have you had any health problems (e.g. memory loss, seizures, hepatitis, bleeding) as a result of your drug use?
